# Improved Thermo-Mechanical Fatigue Resistance of Al-Si-Cu 319 Alloys by Microalloying with Mo

**DOI:** 10.3390/ma16093515

**Published:** 2023-05-03

**Authors:** Kun Liu, Shuai Wang, Peng Hu, Lei Pan, X.-Grant Chen

**Affiliations:** 1 Department of Applied Science, University of Quebec at Chicoutimi, Saguenay, QC G7H 2B1, Canada; kun.liu@uqac.ca (K.L.); swang4@etu.uqac.ca (S.W.); phu@etu.uqac.ca (P.H.); 2 Arvida Research and Development Centre, Rio Tinto Aluminum, Saguenay, QC G7S 4K8, Canada; leiray.pan@riotinto.com

**Keywords:** Al-Si-Cu 319 alloy, thermo-mechanical fatigue, Mo addition, precipitation coarsening, dispersoids, energy-based prediction model

## Abstract

Thermo-mechanical fatigue (TMF) is one of the most detrimental failures of critical engine components and greatly limits their service life. In this study, the out-of-phase TMF (OP-TMF) behavior in Al-Si-Cu 319 cast alloys microalloyed with Mo was systematically investigated under various strain amplitudes ranging from 0.1–0.6% and temperature cycling at 60–300 °C and compared with the base 319 alloy free of Mo. Cyclic stress softening occurred in both experimental alloys when applying the TMF loading, resulting from the coarsening of θ’-Al_2_Cu precipitates. However, the softening rate of the Mo-containing alloy was lower than that of the base 319 alloy because of its lower θ’-Al_2_Cu precipitate coarsening rate per cycle. The Mo-containing alloy exhibited a longer TMF lifetime than the base alloy at the same strain amplitude. Microalloying 319 alloy with Mo enhanced the TMF resistance mainly by slowing the coarsening of θ’-Al_2_Cu precipitates and providing supplementary strengthening from thermally stable Mo-containing α-dispersoids distributed in the Al matrix. The energy-based model was successfully applied for predicting the TMF lifetime with a low life predictor factor, which agreed well with the experimentally measured fatigue cycles.

## 1. Introduction

Thermo-mechanical fatigue (TMF) is increasingly attracting great attention in evaluating the elevated-temperature properties of materials in modern industries, especially for the critical components in automotive and aerospace applications, such as engine parts and gas turbines [1,2,3,4,5,6]. In combustion engine blocks and cylinder heads, TMF could be a result of the cyclic thermal and mechanical load due to the start-up and shutdown of the engines, where a complex change of loading, as well as a dramatic temperature gradient, can occur owing to the different thermal behaviors of various engine components [1,7,8]. Therefore, their service life could be greatly reduced due to the presence of TMF [1,3,9]. For that reason, understanding the TMF mechanism and further improving the TMF resistance of materials plays a significant role in the safety design of these components. Based on the cyclic evolution of load and temperature, TMF is generally categorized into two types: (i) in-phase TMF characterized by the maximum load and highest temperature being reached at the same time, and (ii) the out-of-phase (OP) TMF, in which the maximum load is reached at the minimum temperature. The OP-TMF is reported to be the principal damage mechanism in engine components [1].

Microalloying is a common and applicable approach for achieving the desirable microstructure and mechanical properties of Al alloys, and it can significantly influence the TMF resistance of materials. For example, the TMF behavior of Ni-based superalloys in advanced gas-turbine engines has been widely studied [3,4,5,9]. The moderate addition of Si (0.25 wt.%) doubles the TMF life of a Ni-based single-crystal superalloy at elevated temperatures [5], whereas W, Ta, and Re can also improve TMF resistance by forming W-, Ta-, and Re-rich precipitates [4]. Aluminum cast alloys, such as Al-Si-Cu cast alloys, are widely used in engine parts in the automotive industry to replace cast iron because of their light weight, high strength-to-weight ratio, superior castability, and good thermal conductivity [1,7,10]. Several studies have evaluated the effect of alloying elements on the evolution of TMF resistance in Al-Si cast alloys. Copper has been reported to increase TMF resistance through the formation of Cu-rich precipitates and their better stability during cyclic loading [8,11,12], whereas Sr is beneficial to TMF resistance owing to the modification of the Si structure [11,13,14]. However, Fe has been reported to be detrimental to TMF owing to the formation of brittle Fe-rich intermetallics [10,13,15]. In recent years, it has been found that microalloying Al-Si cast alloys with Mo can remarkably enhance their elevated temperature properties [16,17,18,19,20]. These studies have discovered that both the mechanical strength and creep resistance at 300 °C (approximately 0.65 *Tm*) are significantly improved by the precipitation of thermo-stable Mo-containing dispersoids as well as by modifying the intermetallics. However, few studies have been conducted to explore the effect of Mo on the TMF behavior in Al-Si cast alloys.

Our previous study revealed that the TMF resistance was considerably higher in the Al-Si-Cu 319 alloy than in the Al-Si-Mg 356 alloy [8]. In this study, 319 alloy was microalloyed with Mo to perform OP-TMF tests at different strain amplitudes of 0.1–0.6% with a temperature cycling of 60–300 °C. The TMF behaviors of both experimental alloys, such as the stress-strain hysteresis loop, cyclic evolution of stress, and TMF lifetime, were evaluated. The microstructural evolution before and after TMF cycling was characterized to establish a correlation between the microstructure and TMF behavior. An energy-based model was used for predicting the TMF lifetimes of the two alloys.

## 2. Materials and Methods

**Figure 1:**
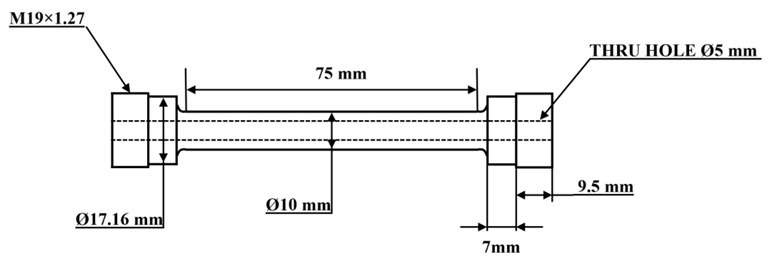
Geometry and dimension of the TMF sample.

Two Al-Si-Cu type 319 alloys, one free of Mo and one with Mo addition (hereafter referred to as “319” for the base alloy and “319M” for the alloy with a 0.3 wt.% Mo addition), were prepared in the laboratory using commercially pure Al (99.7%), pure Mg (99.9%), Al-50%Si, Al-25%Mn, Al-50%Cu, Al-10%Sr, Al-10%Mo, and Al-5%Ti-1%B master alloys. The materials were melted in a clay-graphite crucible using an electric resistance furnace. The details of the casting process can be found in [8,21]. The chemical compositions of the two experimental alloys were analyzed by optical emission spectroscopy, and the results are presented in Table 1. After casting, the samples were heat-treated in T7 condition, which is composed of a two-step solution treatment (495 °C for 4 h + 515 °C for 2 h) followed by water quenching and artificial aging at 200 °C for 5 h. Both solution treatment and aging treatment were performed in an air-forced electric furnace equipped with a programmable temperature controller (±2 °C), and the heating rate was set as 1 °C/min. The T7-samples were machined to a dog-bone shape with a gauge length of 75 mm and a diameter of 10 mm for TMF tests, as shown in Figure 1. A hollow sample with a 5 mm-diameter hole was designed for air cooling.

The OP-TMF tests were conducted on a Gleeble 3800 thermo-mechanical simulator unit in the strain-controlled mode, which is capable of rapid heating/cooling of the sample with controlled heating/cooling rates [21]. The samples were heated using a Joule heating system [21], which produces heat through the sample with an electric current and generates a low radial/axial temperature gradient in the gauge length of the sample, providing fast and uniform heating for reliable results with low deviation; the fast cooling was achieved by passing the compressed cool air through the central hole (Figure 1). The test temperature was measured and controlled with a thermocouple spot-welded at the center of the sample. The strain amplitude for OP-TMF was 0.1–0.6%, and the temperature cycle was 60 to 300 °C during heating and 300 to 60 °C during cooling, with a heating/cooling rate of 5 °C/s for each cycle [8]. Two criteria were applied to automatically terminate the test, namely, when a total of 2000 cycles was achieved or a 30% decrease in the initial maximum tensile/compression stress occurred.

After the installation of the sample and prior to the strain-controlled TMF test on the Gleeble unit, a pre-test was performed, which consisted of Young’s modulus measurements, multi-cycle measurements of thermal strain, and zero-stress adjustment [6]. Young’s modulus was measured at T_min_ and T_max_ three times. To determine the thermal strain as a function of temperature, a number of thermal cycles (approximately 10–15) were performed in the stress-controlled mode at near-zero stress using the same temperature cycle as the actual TMF test. Subsequently, several other thermal cycles (approximately 10) were used for the zero-stress adjustment back in the strain-controlled mode. Further details regarding the test procedure can be found in our previous study [21].

The microstructural evolution under various conditions was analyzed by both optical and transmission electron microscopies. Optical microscopy was used to characterize the as-cast and T7 microstructures. Transmission electron microscopy (TEM) was performed to characterize the evolution of the precipitates and dispersoids before and after TMF testing. TEM samples were acquired from both the solution- and T7-treated samples, as well as from post-TMF samples in the gauge zone close to the fracture face. Then, TEM samples were prepared by mechanical grinding and polishing to ~30 μm thickness and were punched into 3 mm diameter disks, followed by twin-jet electrochemical polishing in a solution of 75 mL HNO_3_ in 250 mL methanol at −30 °C under the voltage of 20 V and current of 20–25 mA. All TEM images were captured along the <001>Al zone axis. Image analysis was applied to quantify the characteristics of the precipitates and dispersoids, such as their size, area fraction, and number density.

## 3. Results

### 3.1. T7-Treated Microstructures

**Figure 2:**
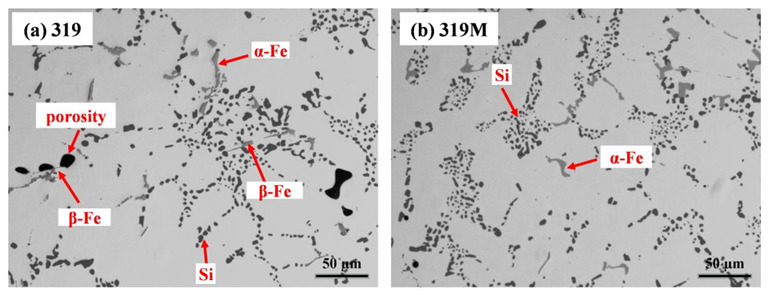
Microstructures of two alloys under T7 condition, (**a**) 319 and (**b**) 319M.

Figure 2 shows the microstructures of the T7-treated experimental alloys, which contained an Al matrix, spheroidized eutectic Si particles, and two kinds of Fe-rich intermetallics. The primary Mg_2_Si and θ-Al_2_Cu formed in the as-cast condition mostly dissolved back in the Al matrix during the solution treatment. However, both needle-like β-Fe (Al_5_FeSi) and Chinese-script α-Fe (Al_15_(FeMn)_3_(SiCu)_2_) coexisted in 319 (Figure 2a) with β-Fe and α-Fe area fractions of 0.4% and 1.2%, respectively, whereas only α-Fe was observed in 319M (Figure 2b), which resulted from the neutralization effect of Mo on Fe-rich intermetallics [19]. Owing to the addition of Mo, the area fraction of the total Fe-rich intermetallics was slightly higher in 319M (1.9%) than in 319 (1.6%). Additionally, some porosities were observed close to the needle-like β-Fe in 319 (Figure 2a), and the total area fraction of porosity in 319 was slightly higher than that in 319M, which could be due to the fact that needle-like β-Fe can be a potential nucleus for porosity during the solidification process [22,23].

**Figure 3:**
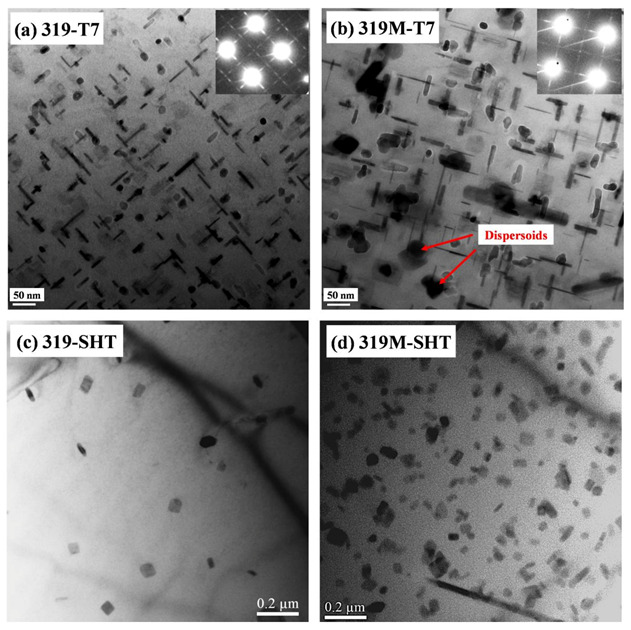
Bright-field TEM images showing the precipitates and dispersoids in the experimental alloys: (**a**,**b**) θ’-Al_2_Cu in 319 and 319M after T7 aging and (**c**,**d**) α-dispersoids in 319 and 319M after solution treatment.

In addition to the difference between the intermetallics in the two alloys, there were also precipitation differences in the Al matrix. The Al-Cu-Si 319 alloy is a heat-treatable and precipitation-strengthening alloy. The bright-field TEM images in Figure 3 show the precipitation microstructure after the T7 treatment but before the TMF test. Figure 3a,b show that the major precipitates in both alloys were θ’-Al_2_Cu, which are identified by the selected area’s diffraction pattern inserted in the images. The θ’-Al_2_Cu precipitates in 319 (Figure 3a) were slightly finer, with a higher number density than that in 319M (Figure 3b). As shown in Table 2, the length of the θ’-Al_2_Cu precipitates in 319 was 48 nm with a width of 4.2 nm, as compared with that of 65 nm (length) and 5.4 nm (width) in 319M. The number densities of θ’-Al_2_Cu precipitates in 319 and 319M were 4251 and 3953 μm^−1^, respectively. The lower number density of coarser θ’-Al_2_Cu precipitates in 319M can be explained by the higher area fraction of Fe-rich intermetallics (α-Al_15_(FeMn)_3_(SiCu)_2_) that contain Cu in 319M, as compared with the Cu-free β-Al_5_FeSi in 319 [19]. Therefore, some Cu solutes could be consumed in the intermetallics, leading to the less Cu being available to form the θ’-Al_2_Cu precipitates in 319M.

The formation of dispersoids in the two alloys also differed, as shown in Figure 3c,d. Because both alloys contained Fe and Mn or Mn/Mo, dispersoids were generally formed during solution treatment and remained after T7 aging and during TMF cycling because of their high thermal stability [16,17,20]. Only a few dispersoids were sparsely distributed in the Al matrix of 319 (Figure 3c), whereas a high number density of dispersoids could be observed in 319M (Figure 3d). According to the TEM-EDS results, the dispersoids in 319 and 319M contained Fe, Mn, and Si and Fe, Mn, Mo, and Si, respectively. Despite the difference in the dispersoid chemistry resulting from the Mo addition, all dispersoids were identified as the same type of α-dispersoids (AlFeMnSi/AlFeMnMoSi) according to the selected area’s diffraction patterns (not shown here) and information in the literature [17,20]. As shown in Table 2, the area fraction of the dispersoids in 319M was significantly higher than that in 319 (3.1% and 0.4%, respectively). This can be attributed to the synthetic effect of the combined addition of Mn and Mo, which significantly enhances the formation of dispersoids [17,20,24]. Because dispersoids are formed during solution treatment, they can act as nuclei for the θ’-Al_2_Cu precipitates during the subsequent aging treatment [25]. Figure 3b shows an image of a mixture of θ’-Al_2_Cu precipitates and α-dispersoids in 319M.

### 3.2. TMF Behaviors

**Figure 4:**
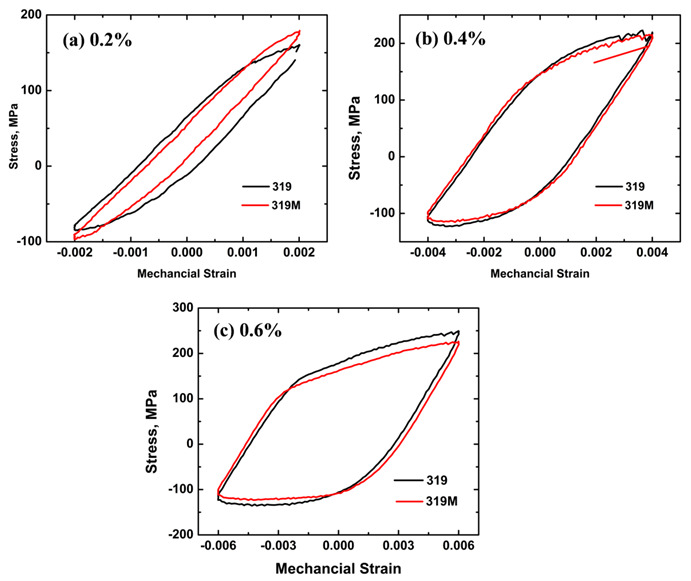
Second hysteresis loops of the two alloys at strain amplitudes of (**a**) 0.2, (**b**) 0.4, and (**c**) 0.6%.

In the present work, OP-TMF was performed for two alloys under strain amplitudes of 0.1, 0.2, 0.4, and 0.6%. In the case of the 0.1% strain amplitude, both alloys trigged the limited cycles (2000 cycles) to stop the test, which may not represent the real TMF lifetime. Therefore, the focus was put on TMF behaviors under 0.2, 0.4, and 0.6%, in which the TMF lifetimes were below 2000 cycles. Figure 4 shows the stress-strain hysteresis loops of the second cycles of two alloys, which present the initial condition of the sample during TMF cycling. Both alloys exhibited some similarities in the hysteresis loops. First, the hysteresis loops under all strain amplitudes were asymmetric, especially under higher strain amplitudes (0.4% and 0.6% in Figure 4b,c). It displays a higher portion in tensile than in compression, which could be derived from the characteristics of OP-TMF, where the maximum tensile strain was reached at the lowest temperature (60 °C) and the maximum compression strain occurred at the highest temperature (300 °C). It is well known that the strength of Al-Si cast alloys decreases with increasing temperature [16,20,26]. Hence, during one cycle, the tensile stress is always higher, owing to its low temperature, compared with the compression stress at a high temperature under the same tensile/compression strain. Second, the peak tensile or compression stresses increased with increasing strain amplitudes, which can be explained by the mechanical behavior of alloys that demonstrates more stress is required to reach higher strain. Taking 319M as an example, the peak tensile stress increased from 176 MPa at 0.2% to 213 MPa at 0.4% and further to 223 MPa at 0.6%.

Differences in the hysteresis loops of the two alloys were also observed in Figure 4. Figure 4a shows that the maximum tensile/compressive stress in 319M was higher than that in 319 at a strain amplitude of 0.2%. As mentioned above, numerous Mo-containing α-dispersoids formed in 319M together with θ’-Al_2_Cu precipitates, resulting in the higher mechanical properties of 319M than that of 319 (130 HV vs. 112 HV after T7), which is consistent with the literature that reveals Mo-containing dispersoids improve the mechanical properties of 319 alloys [20]. Therefore, it is reasonable to expect a higher maximum tensile/compressive stress in 319M during TMF cycling.

However, as shown in Figure 4b, the hysteresis loops of the two alloys at a strain amplitude of 0.4% almost overlapped, indicating similar maximum tensile/compressive stresses for both alloys. Furthermore, the maximum tensile/compressive stress in 319M at a strain amplitude of 0.6% was lower than that in 319 (Figure 4c). The reason for this is the different number of cycles in the pre-tests. As mentioned in the experimental section, to measure the thermal strain and adjust the zero stress, thermal cycling in the temperature range of 60–300 °C was necessary, which could be considered as an “over-aging” process for θ’ precipitates. Table 3 lists the number of cycles of the pre-test and the maximum tensile stress of the second cycle under various strain amplitudes. The number of cycles for the pre-tests at the strain amplitudes of 0.1 and 0.2% was similar for both alloys (approximately 27–29 cycles), indicating a similar “over-aging” process. Therefore, the maximum tensile stress of 319M at the strain amplitudes of 0.1 and 0.2% was higher than that of 319. However, the number of cycles of the pre-test for 319M at a 0.4% strain amplitude was higher than that of 319 (31 and 26, respectively), and significantly higher at a 0.6% strain amplitude (39 and 28, respectively), which resulted in a longer “over-aging” process in 319M, and hence, the lower tensile stress when the TMF tests started.

**Figure 5:**
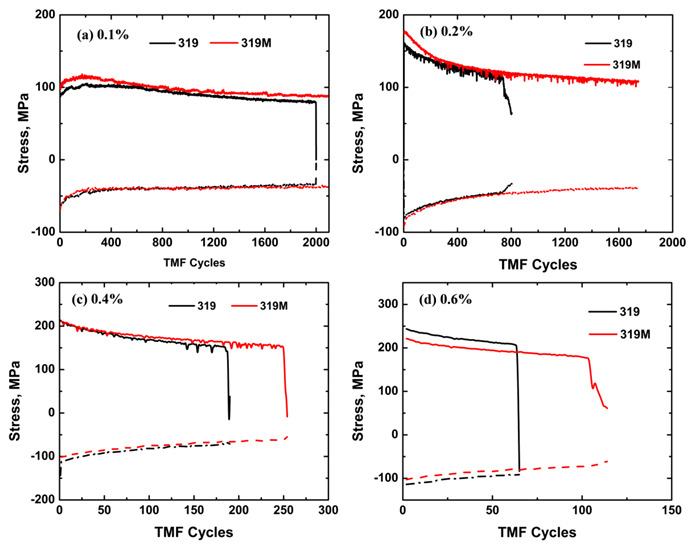
Evolution of the maximum tensile and compressive stresses of two alloys with fatigue cycles under the strain amplitudes of (**a**) 0.1%, (**b**) 0.2%, (**c**) 0.4%, and (**d**) 0.6%.

Figure 5 displays the change of maximum tensile and compressive stresses of the two alloys with the fatigue cycles under various strain amplitudes. Similar to the results in Table 3, the initial maximum stress in 319M was higher at the 0.1 and 0.2% strain amplitudes (Figure 5a,b); however, it was higher in 319 at 0.6% (Figure 5d). Additionally, the tensile and compressive stresses generally decreased with an increasing number of cycles, indicating cyclic softening [27,28]. However, the softening rate was lower in 319M than in 319. Taking the 150 cycles at a strain amplitude of 0.4% (Figure 5c) as an example, both alloys started with similar stresses (213 and 211 MPa in 319 and 319M, respectively), but the stresses after 150 cycles were 158 and 167 MPa for 319 and 319M, respectively, which translates to an average softening rate of 0.37 and 0.29 MPa/cycle in 319 and 319M, respectively. This behavior is strongly related to the coarsening of the precipitates during TMF cycling, as discussed in Section 4.1.

**Figure 6:**
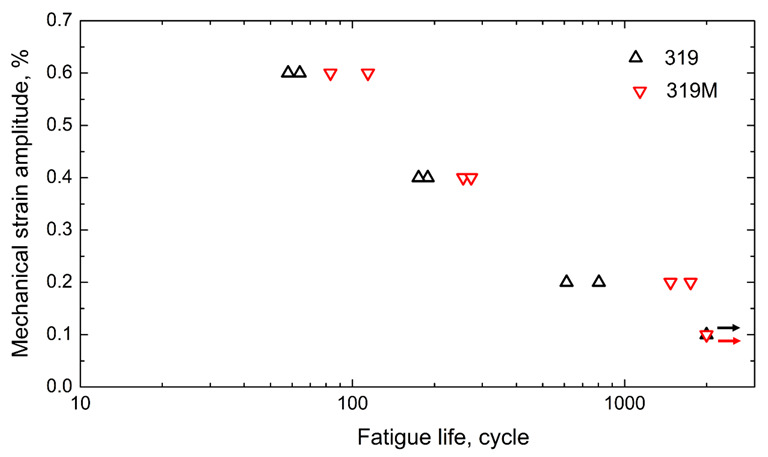
TMF lifetimes of two experimental alloys at different strain amplitudes.

Figure 6 shows the TMF lifetimes of experimental alloys under different strain amplitudes. Generally, the total fatigue life decreases with an increasing strain amplitude. However, the fatigue life of 319M was always longer than that of 319. As shown in Figure 6, the fatigue lives of both alloys exceeded the 2000 cycle limit at a 0.1% strain amplitude; however, the difference became obvious starting at the 0.2% strain amplitude. The average TMF life for 319 at a 0.2% strain amplitude was 708 cycles, which more than doubled to 1611 cycles for 319M. The average TMF lives of 319 and 319M at the strain amplitudes of 0.4 and 0.6% were 182 and 264 cycles, and 61 and 98 cycles, respectively, which are improvements of 46% at 0.4% and 61% at 0.6% in 319M, as compared with 319. It is apparent that the microalloying with Mo significantly improved the TMF resistance of 319 alloys.

## 4. Discussion

### 4.1. Microstructure Evolution during TMF and the Role of Mo in Enhancing TMF Resistance

**Figure 7:**
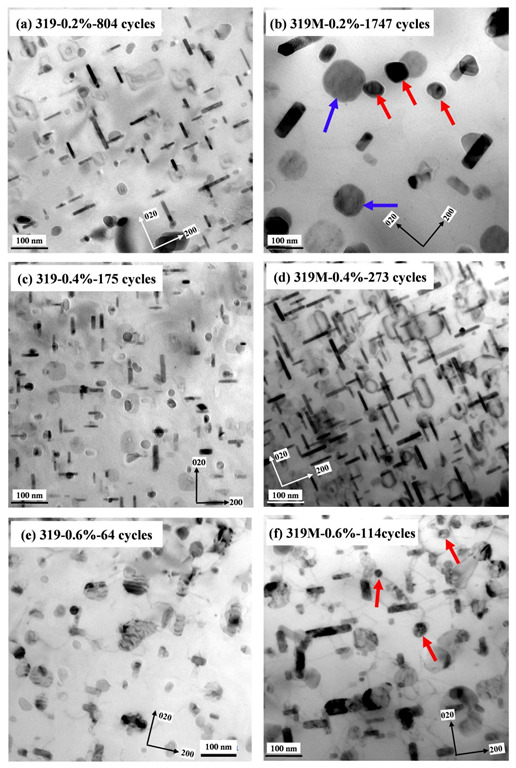
Distribution of θ’-Al_2_Cu precipitates and α-dispersoids after TMF testing under the strain amplitudes of (**a**,**b**) 0.2%, (**c**,**d**) 0.4%, and (**e**,**f**) 0.6%. Blue arrows indicated equilibrium θ-Al_2_Cu and red arrows indicated α-dispersoids.

As shown in Figure 5, the maximum tensile/compressive stress decreased gradually with an increasing number of cycles for both experimental alloys at all applied strain amplitudes. This softening behavior is most likely related to the coarsening of the precipitates during TMF cycling. The alloys exhibited almost no change in the intermetallic compounds and dispersoids after TMF testing. However, the θ’-Al_2_Cu precipitates in both alloys underwent coarsening due to the effect of cyclic temperature and stress. Figure 7 shows the θ’-Al_2_Cu precipitations in the experimental alloys after TMF tests at the strain amplitudes of 0.2, 0.4, and 0.6%. Table 4 provides the quantitative results of θ’-Al_2_Cu precipitates after TMF testing. Compared with Figure 3 and Table 2, under the T7 condition, the θ’-Al_2_Cu precipitates after TMF testing elongated and thickened and exhibited a lower number density at all strain amplitudes. In particular, under a low strain amplitude of 0.2%, the θ’-Al_2_Cu precipitates in 319M severely coarsened and some even transformed into equilibrium θ-Al_2_Cu (indicated by blue arrows in Figure 7b). Figure 7 also shows that the coarsening of θ’-Al_2_Cu precipitates varied with the alloys. For example, as shown in Figure 7e,f, although the number of cycles of 319M under a strain amplitude of 0.6% was double that of 319 (114 vs. 64), the coarsening of the θ’-Al_2_Cu precipitates was even less severe in 319M than in 319. As summarized in Table 4, the length of the θ’-Al_2_Cu precipitates in 319M was 78 nm, which was slightly shorter than the 80 nm in 319. Similar to the strain amplitude of 0.4% (Figure 7c,d), the length and width of the θ’-Al_2_Cu precipitates in 319M after 273 cycles were similar to those in 319; however, the number of cycles in 319 was lower (175 cycles).

Due to the large difference in the number of cycles after TMF testing, the different coarsening rates of θ’-Al_2_Cu in the two alloys could hardly be compared directly from Figure 7 and Table 4. To appropriately evaluate the coarsening rate of θ’-Al_2_Cu precipitates during TMF cycling, which is the main strengthening phase in 319 alloys, the classical Lifshitz–Slyozov–Wagner (LSW) model, as expressed in Equation (1) [29,30,31], was applied.

(1) $$L^{n}-L_{0}^{n}=k\cdot t$$

where *L*_0_ and *L* are the average half-lengths of the θ’-Al_2_Cu before and after TMF tests, respectively, and *k* is the coarsening rate constant, while *n* is the temporal exponent, which is assumed to be 2 in this study, according to the literature [32,33]. *t* is normally presented as time in the classical LSW model because the coarsening of precipitates is often treated under isothermal conditions [30,31]. However, in this study, the TMF test experienced cyclic changes in both temperature and stress, making it difficult to evaluate the coarsening rate when considering only the time factor. For simplicity, *t* was modified to the TMF cycles and then *k* was calculated as the coarsening rate per cycle in the present work. Using the data in Table 2 and Table 4, the coarsening rate per cycle of the θ’-Al_2_Cu precipitates in the two alloys was calculated and summarized in Table 5.

As shown in Table 5, the *k* value as the coarsening rate per cycle of the θ’-Al_2_Cu precipitates for both alloys generally increased with the increasing strain amplitude, indicating more severe coarsening of the precipitates at higher strain amplitudes. At high strain amplitudes, severe deformation was introduced into the alloys, generating heavy dislocations in the Al matrix, which promoted the diffusion of elements and contributed to the high coarsening rate of the precipitates [34,35]. However, 319M exhibited a significantly lower coarsening rate than 319 at a given strain amplitude. For instance, the value of *k* in 319M at a strain amplitude of 0.6% was only 4.2, as compared with 16 in 319. The coarsening rates in 319M at the strain amplitudes of 0.2, 0.4, and 0.6% were only 51, 58, and 27% of those of 319, respectively (Table 5).

As shown in Figure 3, microalloying with Mo promoted the formation of a large number of Mo-containing α-dispersoids in the Al matrix. These dispersoids were thermally stable up to 350 °C [16,24] and were retained in the matrix with little change in the size and number density during TMF cycling (see red arrows in Figure 7). It has been reported that Mo-containing dispersoids can enhance the coarsening resistance of θ’-Al_2_Cu precipitates by forming an elastic strain field and inhibiting dislocation motion [34,35]. Therefore, the formation of thermally stable Mo-containing dispersoids in 319M also plays a significant role in the coarsening behavior of the θ’-Al_2_Cu precipitates during TMF cycling. The low coarsening rate of the θ’-Al_2_Cu precipitates in 319M resulted in a lower decrease in mechanical stresses, and hence, a higher TMF life. On the other hand, the stable Mo-containing dispersoids in 319M provided supplementary strengthening to the Al matrix, particularly at high temperatures. It has been reported that α-dispersoids can significantly trap dislocations during high-temperature deformation and produce homogeneous deformation, resulting in a reduction in the local stress concentration [36]. Although the Mo-containing dispersoids were relatively bigger than the initial θ’-Al_2_Cu precipitates, the θ’-Al_2_Cu precipitates gradually coarsened with an increasing number of cycles, and the strengthening effect of the Mo-containing dispersoids became more significant, further contributing to the high TMF life of 319M.

In addition to these two major influences on enhancing TMF resistance, the addition of Mo to 319M also resulted in a complete transformation from needle-like β-Fe to Chinese-script α-Fe (Figure 2). Needle-like β-Fe intermetallics generally cause local stress concentrations in the matrix and accelerate crack propagation during loading [37,38]. Therefore, the Chinese-script intermetallics in the 319M alloy can reduce crack propagation during TMF, and hence, improve the fatigue life.

### 4.2. Prediction of TMF Lifetime

**Figure 8:**
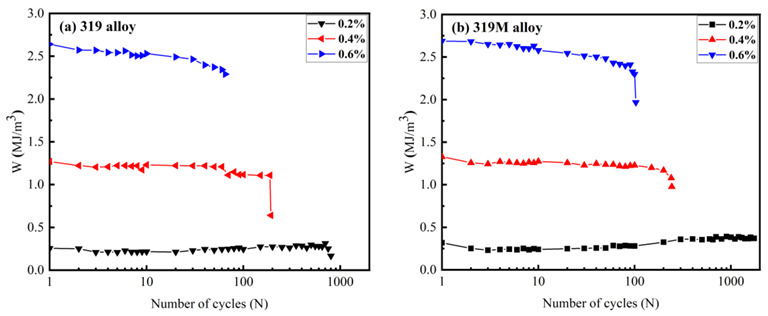
Evolution of the hysteresis energy (W) with cycles for (**a**) 319 and (**b**) 319M.

In general, TMF testing is a complex test technique that requires higher skills and more reliable testing equipment than traditional mechanical testing [6]. It normally takes a very long time to perform a series of TMF tests under various strain amplitudes to evaluate the TMF resistance of materials. Therefore, it is of significance to predict and verify TMF lifetimes with limited data. However, TMF lifetime is difficult to accurately predict because the temperature and stress continuously change during TMF cycling. Several models based on different mechanisms have been proposed, including but not limited to the J-integral model with fracture mechanics [39]; the Miller model for the accumulation of the damage rate [40]; the Neu-Sehitoglu model, using fatigue, creep, and oxidation damage [41]; and energy-based models derived from the evolution of dissipated energy per cycle [42,43,44,45]. The energy-based model has proven to be a more suitable approach for predicting TMF lifetime [42,46] owing to the introduced hysteresis energy, which reflects the change in both stress and strain amplitudes. The hysteresis energy is approximately calculated as the product of the stress range (Δ*σ*) and plastic strain range (Δ*ε_p_*), as shown in the following Equation (2) [8,42]:

(2) $$W_{i}=\int \sigma d\varepsilon \approx \Delta \varepsilon_{p}\Delta \sigma$$

where *W_i_* denotes the hysteresis energy of the *i*th cycle. Figure 8 shows the hysteresis energy (*W*, equal to the hysteresis loop area fraction) as a function of the number of cycles for both 319 and 319M. The hysteresis energy stabilized and saturated after several cycles at the applied strain amplitudes. Therefore, the saturation hysteresis energy could be easily estimated from the initial few cycles.

The saturated hysteresis energy (*W_s_*, also called the plastic strain energy) is closely related to the fatigue life (*N_f_*), as expressed by Equation (3) [42]:

(3) $$W_{s}=W_{0}N_{f}^{-1/\beta }$$

where *W*_0_ and *β* are the material parameters reflecting the fatigue damage capacity and fatigue damage exponent, respectively [42]. Both *W*_0_ and *β* can be calculated from the log*W_s_* − log*N_f_* relationship.

**Figure 9:**
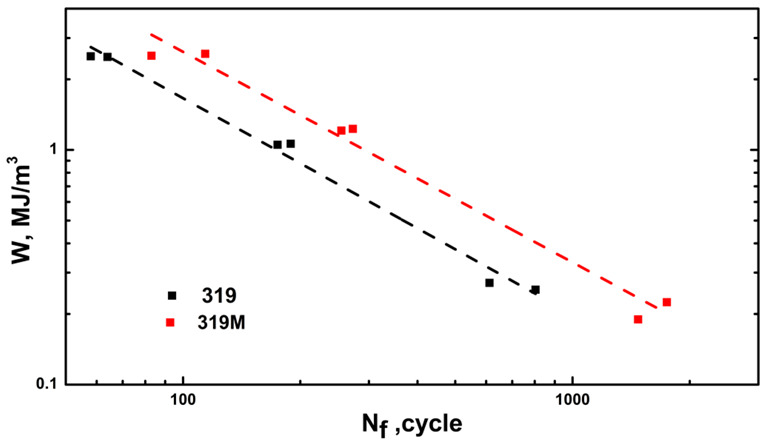
Relationship between *W* and *N_f_* in the two experimental alloys.

Based on the experimental data of the fatigue life in Figure 6 and the saturated hysteresis energy (*W_s_*) in Figure 8, their relationship is shown in Figure 9 on a log-log scale, and the calculated *W*_0_ and *β* values are listed in Table 6. The slopes of the fitting curves for 319 and 319M were similar, indicating similar *β* values for both alloys. As shown in Table 6, *β* was calculated to be 1.09 and 1.07 for 319 and 319M, respectively, indicating similar decreasing TMF cycles with hysteresis energy. However, the intersections of the fitting curves with the *Y*-axis for the two alloys, from which *W*_0_ was calculated, were considerably different. As shown in Table 6, the *W*_0_ of 319M was 201, which is significantly higher than the 108 of 319, indicating the high fatigue damage capacity of 319M, hence the enhanced TMF resistance.

**Figure 10:**
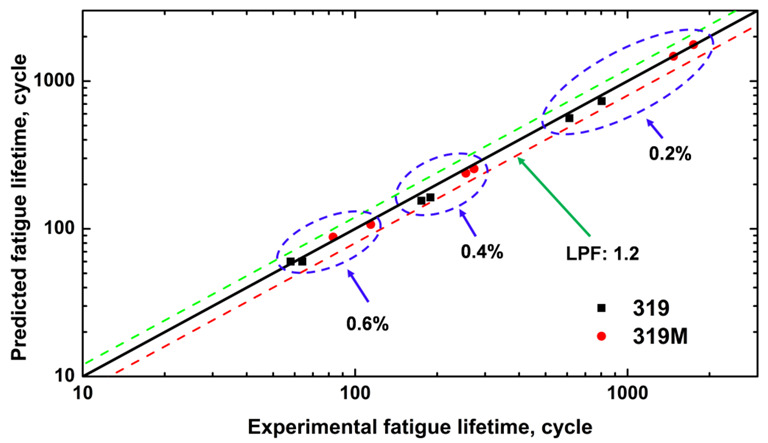
Comparison of the predicted and experimental TMF lifetime of the two alloys.

The predicted fatigue lifetimes of the two alloys were calculated using Equation (3) and the material parameters (*W*_0_ and *β*) in Table 6. The life prediction factor (LPF), which is defined as the ratio of the predicted life to the experimental life, is often used to evaluate the applicability of prediction models. Figure 10 shows the predicted and experimental TMF lifetimes for the two alloys with relatively low LPF (approximately 1.2). The results demonstrate that the predicted lifetimes agree well with the experimentally measured ones, confirming that the energy-based model is applicable for predicting the TMF lifetime of Al-Si-Cu 319 alloys. Using this prediction, the TMF lifetimes of 319 and 319M under a 0.1% strain amplitude were estimated to be 4152 and 7382 cycles, respectively, both were remarkably longer than those of the non-fractured samples tested for 2000 cycles.

TMF testing is generally time-consuming, particularly at low strain amplitudes. For example, the fatigue lifetime of 319 at a 0.2% strain amplitude was 1000–2000 cycles, and each cycle duration in this study was approximately 2 min. Additionally, the TMF lifetimes of 319 and 319M at a 0.1% strain amplitude were predicted to be 4152 and 7382 cycles, respectively, which could take approximately 6–10 days of nonstop running tests on the Gleeble unit. TMF lifetime can be measured and predicted to reduce experimental time by using a relatively simple energy-based model. For a given material, TMF tests can be initially performed at higher strain amplitudes, such as 0.4 and higher, which is expected to result in shorter fatigue life and require significantly less experimental time. Subsequently, *W*_0_ and *β* in Equation (3) can be obtained using the measured *W_s_* and *N_f_* from those 2–3 higher strain amplitudes, as shown in Figure 9. On the other hand, as shown in Figure 8, the saturated hysteresis energy (*W_s_*) can generally be reached and calculated after several initial cycles at lower strain amplitudes without waiting until the end of the TMF test. Therefore, the fatigue life of the material under lower strain amplitudes can be appropriately predicted using the known *W*_0_ and *β*. Thus, the experimental time of a TMF test can be significantly reduced to evaluate the TMF resistance of materials by applying an energy-based model.

## 5. Conclusions

(1) Cyclic stress softening occurred in both experimental alloys during the TMF tests; however, the softening rate of the Mo-modified 319M alloy was lower than that of the base 319 alloy.
(2) The TMF lifetime decreased with an increasing strain amplitude. The alloy 319M exhibited a longer TMF lifetime than 319 under the same strain amplitude.
(3) The θ’-Al_2_Cu precipitates in both experimental alloys underwent coarsening during TMF cycling. However, the coarsening rate per cycle of θ’-Al_2_Cu in the 319M alloy was significantly lower than that in the 319 base alloy.
(4) Microalloying the Al-Si-Cu 319 alloy with Mo promoted the formation of a large number of thermally stable Mo-containing α-dispersoids and therefore improved the TMF resistance mainly by slowing the coarsening of θ’-Al_2_Cu precipitates and providing supplementary strengthening to the Al matrix.

## Figures and Tables

**Table 1:** Chemical compositions of the experimental alloys (wt.%).

Alloy	Si	Cu	Mg	Mn	Fe	Ti	Sr	Mo	Al
319	5.93	3.34	0.12	0.28	0.31	0.11	0.011	-	Bal.
319M	6.00	3.50	0.11	0.30	0.30	0.10	0.010	0.3	Bal.

**Table 2:** Characteristics of the precipitates and dispersoids under the T7 condition.

θ’-Al_2_Cu	Length, nm	Width, nm	Number Density, μm^−1^
319	48	4.2	4251
319M	65	5.4	3953
**Dispersoids**	**Equivalent diameter, nm**		**Area fraction, %**
319	55		0.4
319M	43		3.1

**Table 3:** Number of cycles of the pre-tests and maximum tensile stress at the 2nd cycle.

Strain Amplitude, %	0.1		0.2		0.4		0.6	
Alloy	319	319M	319	319M	319	319M	319	319M
Pre-test cycles	27	28	29	28	26	31	28	39
Maximum tensile stress at 2nd cycle, MPa	87.6	96.6	159.3	176.5	215.2	213.4	243.6	223.5

**Table 4:** Characteristics of the θ’-Al_2_Cu precipitates after TMF testing.

Alloys	Conditions (Strain Amplitude and Cycle Number)	Length	Width	Number Density
		(nm)	(nm)	(μm^−1^)
319	0.2%, 804 cycles	115.9	12.1	501
	0.4%, 175 cycles	83.9	10.5	789
	0.6%, 64 cycles	80.1	18.1	691
319M	0.2%, 1747 cycles	131.2	36	131
	0.4%, 273 cycles	90.2	10.9	852
	0.6%, 114 cycles	78.1	19.2	521

**Table 5:** Coarsening rate per cycle (k) of the θ’-Al_2_Cu precipitates under TMF cycling.

Alloy	Strain Amplitude, %		
	0.2	0.4	0.6
319	3.5	6.2	16
319M	1.8	3.6	4.2

**Table 6:** Material parameters calculated with an energy-based model for TMF life prediction.

Alloy	*W*_0_ (MJ/m^3^)	*β*
319	107.75	1.09
319M	200.91	1.07

## Data Availability

Supporting data could be made available upon reasonable request.
